# Flow controlled ventilation in Acute Respiratory Distress Syndrome associated with COVID-19: A structured summary of a study protocol for a randomised controlled trial

**DOI:** 10.1186/s13063-020-04708-1

**Published:** 2020-09-11

**Authors:** Stefan Roehrig, Ali Ait Hssain, Nabil Al Hamid Shallik, Ingi Mohamed A. Elsaid, Salma Faisal Mustafa, Osama A. M. Smain, Ashraf Abdulla Molokhia, Marcus D. Lance

**Affiliations:** 1 Department of Anesthesiology, Intensive Care and Perioperative Medicine, Hamad Medical Corporation (HMC), Al-Rayyan Road, Doha, Qatar; 2 Department of Medical Intensive Care, Hamad Medical Corporation (HMC), Al-Rayyan Road, Doha, Qatar; 3 Department of Medical Education, Hamad Medical Corporation (HMC), Al-Rayyan Road, Doha, Qatar

**Keywords:** COVID-19, randomised controlled trial, protocol, ARDS, Flow Controlled Ventilation, Mechanical ventilation

## Abstract

**Objectives:**

This study aims to demonstrate the positive effects on oxygenation of flow-controlled ventilation compared to conventionally ventilated patients in patients suffering from Acute respiratory distress syndrome (ARDS) associated with COVID-19.We define ARDS according to the “Berlin” definition integrating the oxygenation index (P/F ratio), the level of Positive End Expiratory Pressure (PEEP), radiological and clinical findings.

**Trial design:**

This is a prospective, randomized (1:1 ratio), parallel group feasibility study in adult patients with proven COVID-19 associated ARDS.

**Participants:**

All adult patients admitted to the ICU of Hamad Medical Corporation facilities in Qatar because of COVID-19 infection who develop moderate to severe ARDS are eligible. The inclusion criteria are above 18 years of age, proven COVID-19 infection, respiratory failure necessitating intubation and mechanical ventilation, ARDS with a P/F ratio of at least 200mmHg or less and a minimum PEEP 5cmH2O, BMI less 30 kg/ m2. The following exclusion criteria: no written consent, chronic respiratory disease, acute or chronic cardiovascular disease, pregnancy or need for special therapy (prone position and/or Extracorporeal membrane oxygenation).

**Intervention and comparator:**

After randomisation, the group A patients will be ventilated with the test-device for 48 hours. The settings will be started with the pre-existing-PEEP. The upper pressure will be determined to achieve a tidal volume of 6 ml/kg lean body mass, while the respiratory rate will be set to maintain an arterial pH above 7.2.

In group B, the ventilator settings will be adjusted by the attending ICU team in accordance with lung-protective ventilation strategy.

All other treatment will be unchanged and according to our local policies/guidelines.

**Main outcomes:**

The primary end point is PaO2. As this is a dynamic parameter, we will record it every 6-8 hours and analyse it sequentially.

**Randomisation:**

The study team screens the ventilated patients who fulfil the inclusion criteria and randomise using a 1:1 allocation ratio after consenting using a closed envelope method. The latter were prepared and sealed in advance by an independent person.

**Blinding (masking):**

Due to the technical nature of the study (use of a specific ventilator) blinding is only possible for the data-analysts and the patients.

**Numbers to be randomised (sample size):**

The sample size calculation based on the assumption of an effect size (change in PaO2) of 1.5 SDS in the primary endpoint (PaO2), an intended power of 80%, an alpha error of 5% and an equal sample ratio results in n=7 patients needed to treat. However, to compensate for dropouts we will include 10 patients in each group, which means in total 20 patients.

**Trial Status:**

The local registration number is MRC-05-018 with the protocol version number 3. The date of approval is 14^th^ April 2020. Recruitment began 28th May 2020 and is expected to end in September 2020.

**Trial registration:**

The protocol was registered before starting subject recruitment under the title: “Flow controlled ventilation in ARDS associated with COVID-19” in ClinicalTrials.org with the registration number: NCT04399317. Registered on 22 May 2020.

**Full protocol:**

The full protocol is attached as an additional file, accessible from the Trials website (Additional file 1). In the interest in expediting dissemination of this material, the familiar formatting has been eliminated; this Letter serves as a summary of the key elements of the full protocol.

## Supplementary information

**Additional file 1.** Full Study Protocol.

## Data Availability

The final dataset will be available to the research-team, to the local authorities and upon reasonable request to others after agreement of the local IRB. All data are anonymised and stored safely for five years according to local law.

