# Supplementary material for: Flow controlled ventilation in Acute Respiratory Distress Syndrome associated with COVID-19: A structured summary of a study protocol for a randomised controlled trial
Source: Trials. 2020 Sep 11;21:781. doi: 10.1186/s13063-020-04708-1 (PMC7483072; doi:10.1186/s13063-020-04708-1)
Supplement: Supplementary file 1 — Additional file 1. Full Study Protocol. [file 13063_2020_4708_MOESM1_ESM.pdf]

HMC RESEARCH PROTOCOL

Study Title:	Flow controlled ventilation in ARDS associated with COVID-19
Principal Investigator:<br>Co-investigators	Dr. Marcus D. Lance<br>Dr. Ali AitHssain<br>Dr. Nabil Al Hamid Shallik

Contents

1. Synopsis .... **Error! Bookmark not defined.**
2. Abbreviations and Acronyms .... **Error! Bookmark not defined.**
3. Introduction / Background .... **Error! Bookmark not defined.**
4. Objectives .... **Error! Bookmark not defined.**
5. Variables 2
6. Study Methodology .... **Error! Bookmark not defined.**
7. Study Population and Study Setting/ Location .... **Error! Bookmark not defined.**
8. Study procedures .... **Error! Bookmark not defined.**
   Study Duration and Timelines .... **Error! Bookmark not defined.**
   Informed Consent .... **Error! Bookmark not defined.**
   Risk .... **Error! Bookmark not defined.**
   Bio-Specimens & Sample Collection .... **Error! Bookmark not defined.**
   Outcomes .... **Error! Bookmark not defined.**
   Data Collection, Data Management & Confidentiality .... **Error! Bookmark not defined.**
   Subject Withdrawal/ Withdrawal of Consent .... **Error! Bookmark not defined.**
9. Statistical Consideration and Data Analysis .... **Error! Bookmark not defined.**
10. Adverse Event Reporting .... **Error! Bookmark not defined.**
11. Ethical Consideration .... **Error! Bookmark not defined.**
12. Sponsor, Funding & Collaborator Information .... **Error! Bookmark not defined.**
13. Dissemination of Results and Publication policy .... **Error! Bookmark not defined.**
14. References .... **Error! Bookmark not defined.**
15. Appendices .... **Error! Bookmark not defined.**

1. Synopsis

The pandemic of a newly upcoming viral disease which is associated with COVID-19 puts the whole world's health system under pressure.

Patients suffering from this disease mainly develop respiratory symptoms, which can lead to severe acute respiratory distress syndrome (ARDS) necessitating ICU, admission in 10-20% of the cases admitted to hospital. In addition to these symptoms, patients show lymphopenia, cardiac symptoms and altered coagulation profiles. Although those patients are treated in the ICU the mortality is up to 20% due to multiorgan failure [1, 2]. The aim of this study is to show non-inferiority of flow-controlled ventilation [5, 6, 7] compared to standard (lung protective ventilation) [3, 4].

Methods:

After admission to the ICU, the patients will receive information about the study and informed consent will be taken. Upon reaching the criteria for moderate to severe ARDS (P/F ratio below 200 mmHg and PEEP above 5 cmH2O) the patients will be randomized. In the treatment group (group A) the ultra-thin ventilation tube will be placed through the existing tube. Then flow-controlled ventilation will be applied for 48 hours. In the other group (group B) ventilation will be performed according to the lung protective strategy. All other treatment will be unchanged.

Data-collection will be started 1 hour after initiation of the study.

Primary end point is PaO2.

Participants (all enrolled in ABHATH):

Dr. Marcus Lance, Senior Consultant, Anesthesia, SICU

Dr. Ali Aithssain, Senior consultant, MICU

Dr. Nabil Al Hamid Shallik, Senior Consultant Anesthesia

Dr. Ashraf Abdulla Molokhia, Senior Consultant MICU

Dr. Ingi Mohamed Abbas Elsaid

Dr. Salma Faisal Eltayeb Mustafa

2. Abbreviations and Acronyms

COVID-19: Coronavirus disease 2019

ARDS: acute respiratory distress syndrome

FCV: flow-controlled ventilation

PCV: pressure-controlled ventilation

PEEP: positive endexpiratory pressure

PaO2: arterial oxygen partial pressure

BMI: body mass index

SCC: society of critical care

VCV: Volume Controlled Ventilation

OD: outer diameter

ID: inner diameter

FiO2: inhaled oxygen fraction

PaCO2: arterial carbone Dioxide

FiO2: inhaled oxygen fraction

BGA: blood gas analysis

BNP: brain natriuretic peptide

aPTT: activated, partial throboplastin time

INR: international normalized ratio

PT: prothrombin time

CRP: c-reactive protein

Il-6: interleucine 6

ECMO: extracorporeal membrane oxygenation

SOFA: sequential organ failure score

PIP: pressure during inspiration

HMC-IRB,MRC-05-018,14Apr20-13Apr21

3. Background

The pandemic of a newly upcoming viral disease which is associated with COVID-19 puts the whole world's health system under pressure.

Patients suffering from this disease mainly develop respiratory symptoms, which can lead to severe acute respiratory distress syndrome (ARDS) necessitating ICU admission in 10-20% of the cases admitted to hospital (SCC-guideline). In addition to these symptoms, patients show lymphopenia, cardiac symptoms and altered coagulation profiles. Although those patients are treated in the ICU the mortality there is up to 20% due to multiorgan failure [1, 2]. Currently, there is no proven therapeutic strategy next to symptomatic treatment.

Although the severely ill patients will need intubation and invasive ventilation according to ARDS treatment strategies including low tidal volumes and low end-expiratory pressures, not all patients recover their pulmonary function [3, 4].

Flow control ventilation (FCV) is a recently developed ventilation strategy which allows to keep the intrapulmonary pressures low while achieving optimal gas exchange [5]. It had been proven in animal models to improve pulmonary function and oxygenation [6] and in cases with ARDS [7].

Flow Controlled Ventilation mode is a unique ventilation technique in which inspiration as well as expiration are controlled i.e. actively performed. This is achieved by generating a continuous flow into the patient's lungs during inspiration or a continuous (negative) flow, sucking gasses out of the patient's lungs.

The continuous flow without ventilation pauses, results in linear increases and decreases in intratracheal pressures [Figure 1]. As a result, the mean airway pressure will be higher compared to conventional large bore Volume Controlled Ventilation or Pressure Controlled Ventilation (PCV). Therefore, the bronchiole and alveoli will be kept open during ventilation facilitating oxygen uptake to the blood. Moreover, the continuous gas flow enhances gas mixture in the lungs also improving gas exchange. Altogether, FCV results in more efficient ventilation as compared to conventional ventilation

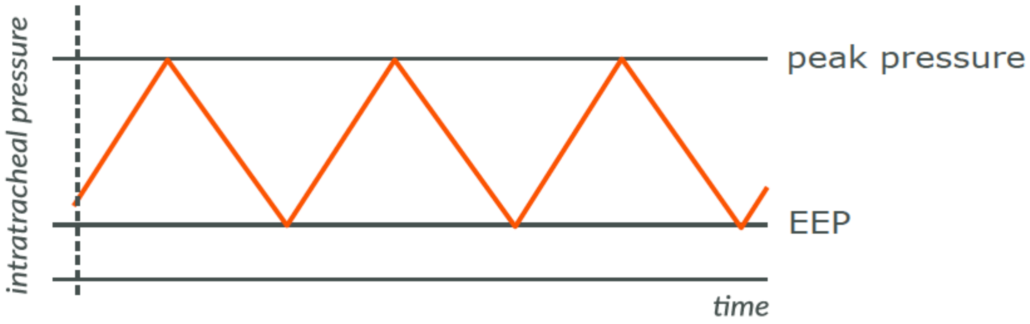

techniques.

Figure 1 pressure volume curve in flow-controlled ventilation

Evone® is the only commercially available ventilator applying FCV ventilation mode, thus directing the inspiration as well as the expiration [Figure 2]. Evone's FCV® ventilation mode is based on a controlled inspiration and expiration flow from a set PEEP to a set peak pressure and vice versa. The inspiratory flow is continuously controlled by advanced mass flow regulators; the expiratory flow is controlled by regulated suctioning.

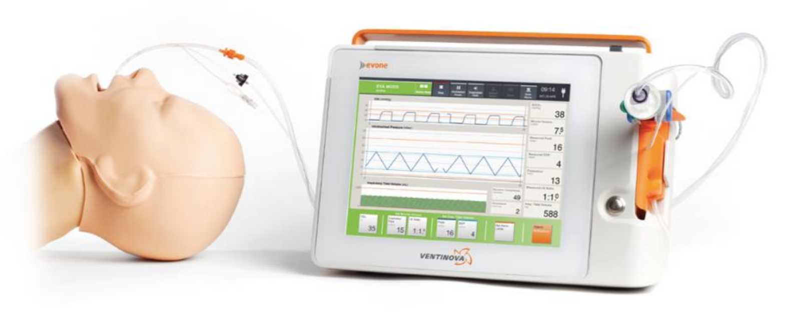

Figure 2: Evone, Ventinova Medical, Eindhoven, The Netherlands

Evone is to be used in combination with Tritube [Figure 3], an ultra-thin endotracheal tube (outer diameter 4.4 mm/ inner diameter 2.4 mm), enabling highly accurate intra-tracheal pressure measurements and securing the airway with an inflatable high volume – low pressure cuff because FCV ventilation requires a sealed airway. With an outer diameter (OD) of only 4.4 mm, Tritube® is an ultra-thin ventilation tube, intended to obtain endotracheal access to the airway and to ventilate an adult patient [Figure 3]. Tritube has three lumina: a ventilation lumen - with Murphy eye and an inner diameter (ID) smaller than 3 mm; a cuff lumen - to inflate and deflate the high volume, low pressure cuff; and an intra-tracheal pressure measurement lumen - for continuous intra-tracheal pressure measurements. Tritube (including its cuff) is completely manufactured of Polyurethane. Additionally, Tritube has a malleable stylet to facilitate intubation [8, 9].

Aim of the study

This study aims to demonstrate the positive effects on oxygenation of flow-controlled ventilation compared to conventionally ventilated patients (pressure control ventilation) in patients suffering from ARDS associated with COVID-19.

HMC-IRB,MRC-05-018,14Apr20-13Apr21

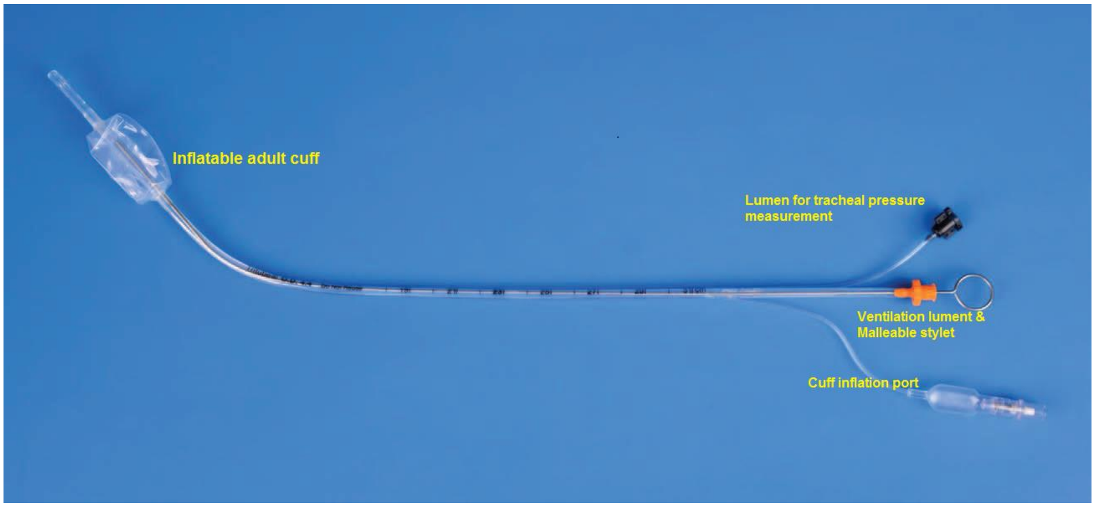

Figure 3: Tritube, Ventinova Medical, Eindhoven, The Netherlands

4. Objectives

Primary outcome:

We choose as the primary endpoint arterial oxygen partial pressure (PaO2).

Secondary Outcomes:

- Minute volume
- Tidal volume
- Respiratory rate
- Intertidal tracheal pressure amplitude
- Peak airway pressure
- End-tidal CO2
- Chest wall compliance
- Respiratory resistance
- Inhaled oxygen fraction (FiO2)
- Arterial CO2 (PaCO2)
- Tube obstruction by secretion

5. Variables

We will collect demographic data (age, sex, BMI), co-morbidities and clinical data (need for special intervention, e.g. "proning") drugs used and fluids administered.
Next to the vital parameters (blood pressure, heart frequency and body temperature) the oxygenation parameters (BGA) will be collected 6-8 hourly according to clinical standard.
Kidney-function parameters (creatinine, urea), liver function tests and whole blood counts and immune-parameters like interleukin 6 (IL-6) will be recorded.
Cardiac enzymes will be collected (troponin, BNP) will be evaluated and reported on clinical need.
All of these parameters are reported routinely on daily base for clinical purpose.

Demographic parameters:

- Age
- Gender
- Body Mass Index (BMI)
- Co-morbidities
- Drug history

Laboratory parameters (routinely sampled):

- Coagulation parameters (aPTT, INR/PT, fibrinogen levels, D-dimer)
- Whole blood count (including differential blood count)
- Metabolic parameters (Creatinine level, LDH, liver function tests, albumin level)
- Lactate level
- CRP, Il-6
- Troponin

Clinically parameters:

- Timing of clinical course (e.g. admission, ICU admission, intubation, ECMO)
- Time of intubation
- Time to extubation
- SOFA score

The following respiratory parameters will be recorded together with the BGA (6-8 hourly):

- Pressure during inspiration (PIP)
- Positive End-Expiratory Pressure (PEEP)
- Lung compliance
- Minute volume
- Tidal volume
- Respiratory rate (RR)
- Intratracheal pressure amplitude
- Peak airway pressure
- End-tidal CO2
- Chest wall compliance
- Respiratory resistance
- FiO2

- I: E ratio
- ABG

6. Study Methodology:

This is a prospective, randomized feasibility study in adult patients with proven COVID-19 associated ARDS. We define ARDS according to “Berlin” definition integrating the oxygenation index (P/F ratio), the level of PEEP and clinical findings [3, 4].

We will use an envelope method to randomize patients into the flow-control ventilation group (group A) while all other patients will receive standard treatment (pressure control ventilation-group B). Due to the nature of the investigation (we use a special device and tube) blinding of the study groups will not be possible. The study will be performed in HMC facilities hosting COVID-19 patients.

After admission to the ICU for COVID-19 associated respiratory disease, the patients (or their relatives) will be approached, informed about the study and if agreed informed consent will be taken. Upon intubation the severity of the disease will be assessed thrice daily. When the patient fulfils the criteria for moderate to severe ARDS (P/F ratio between 100mmHg and 200 mmHg with PEEP of minimal 5 cmH2O and below 100mmHg with a minimal PEEP 5 cmH2o, respectively) randomization will be performed. Hereafter the group A patients will receive the ultra-thin Tritube through the existing tube. This does not need exchange of the tube, so it does not generate additional risks for the staff in terms of aerosols.

Group A: Flow-controlled ventilation

For the safety of the staff, only the investigators will be in the room and perform the change to the Tritube. This will be done under full PPE.

Via the existing tube the ultra-thin Tritube will be inserted. For this purpose, the existing tube will be clamped, and the ventilator will be set on hold. Then the connector with the inlet for the tritube will be connected and the Tritube will be inserted. Hereafter, the clamp will be removed, and the ventilator will be re-started. Then the Tritube will be advanced, blocked and connected to the Evone-ventilator. The ventilator settings will be started with the pre-existing-PEEP and the standard ventilator will be set on hold. The upper pressure will be determined to achieve a tidal volume of 6 ml/kg lean body mass, while the respiratory rate will be set to maintain an arterial BGA?? above 7.2 pH.

Group B: standard care (pressure-controlled ventilation)

In this group the ventilator settings will be adjusted by the ICU team (respiratory therapist) in agreement with lung-protective ventilation strategy [3a,4].

All other treatment will be unchanged and according to our local policies/guidelines.

In all groups the first clinical data collection starts after 1 hour of initiation of the “new” ventilation strategy.

The study ends at 48 hours after insertion of the Tritube or if the patient deteriorates needing prone-positioning or ECMO therapy.

7. Study population and study setting

All adult patients admitted to the hospital (HMGH, HGH) because of COVID-19 infection who develop a moderate to severe ARDS are eligible.

Inclusion criteria

- Age above 18 years
- Proven COVID-19 infection
- Respiratory failure necessitating intubation and mechanical ventilation
- ARDS with a P/F ratio of at least <200mmHg and a PEEP of at least >5cmH2O
- BMI less 30 kg/ m2

Exclusion criteria

- Refuse to sign the consent
- Chronic Respiratory disease
- Acute or chronic Cardiovascular disease
- Pregnancy
- Need for special therapy (prone position and/or ECMO)

We want to ensure there is no risk for our patients. Therefore, we propose a review of our first 4 patients regarding quality and safety by a DMSB. If the IRB agrees we could provide names of intensive care doctors experienced in research.

8. Study procedures

Study duration and timelines

Expected duration of the study:6 months

Start time: ASAP (April 2020)

Data collection: 1 month

Data-analysis: 3 months

Preparing a publication: 2 months

Collection of data:

Demographic data, laboratory data, vital parameters and clinical data will be collected via the electronic patient chart (CERNER).

We will maintain privacy for the subject throughout data collection by carrying out data collection in private rooms. Data confidentiality will be maintained by the use of study IDs rather than any identifying data.

All data will be entered into a secure database, which is password protected with restricted access, only assigned by the research team. Each subject will be assigned an alphanumeric study ID, to ensure data confidentiality. The link between the identifier and the study code will be deleted at the end of the study and the anonymized data set will be kept for at least 5 years after study completion per MRC policy.

Informed consent:

After explaining the project details, a written informed consent will be obtained from patient/ or family member if the patient is not able to give consent. The family member chosen for the consent will be the one who is patient's next of kin.

Subject Withdrawal/ Withdrawal of Consent

Subjects can withdraw from the study at any point and this will not be held against them. They will be informed during the consent process of this and will be asked to contact the research team so that the investigator can withdraw them from the study. If a subject should withdraw, the data and samples collected will be destroyed, unless they have already been analyzed or processed or coded.

9. Statistical Consideration and Data Analysis

Power-calculation: In order to keep the total number of patients small we will match the patients according to age, co-morbidities inhaled oxygen fraction and we regard this investigation as a feasibility study. Therefore, the sample size calculation based on the assumption of an effect size (change in PaO2) of 1.5 SDS in the primary endpoint (PaO2), an intended power of 80%, an alpha error of 5% and an equal sample ratio results in n=7 patients needed to treat. However, to compensate for dropouts we will include 10 patients in each group, which means in total 20 patients.

Data analysis: Categorical data will be presented as number and percentage, while interval data will be presented by median and interquartile range (IQR). Normally distributed data will be analyzed by using two-tailed unpaired Students t-test. Continuous variables with skewed distribution will be analyzed using Mann-Whitney U testing and dichotomous variables by means of Fisher's exact test. A p-value <0.05 is considered significant. All data analyses will be done using SPSS version v26 (IBM Corp, Armonk NY, USA). Graphs will be constructed using GraphPad Prism (GraphPad Prism version 5.0a for Windows, GraphPad Software, San Diego CA, USA).

10. Adverse Event Reporting

Although we do not expect adverse events we will follow and report all adverse effects to ensure safety for the patients.

11. Ethical Consideration

- The study will only be conducted after review and approval from MRC and ethical committee of IRB.
- All participating patients/or patient’s family member will be asked for informed consent after explanation of the project and giving written information.
- There will be no change in treatment plan with the exception of the ventilation.
- We will maintain privacy for the subject throughout data collection by carrying out data collection in private rooms or areas that are partitioned.
- Data confidentiality will be maintained by the use of an individual study ID which is stored in a key file together with the identifier separately and safely. All collected data will be stored under the nominator of the study ID. All information is stored in a secured computer file in a locked office in HGH.

- All research is done under recognition of the Helsinki declaration and under full adherence to the MoPH regulations in Qatar.

12. Sponsor, Funding & Collaborator Information

We rely on our own staff and devices HMC financial support for additional devices might be needed.

13. Dissemination of Results and Publication policy

We are planning to present this study in local or international conference and published it in indexed journal (not decided) after completion of study.

14. References

1. Onder G, Rezza G, Brusaferro S. Case-Fatality Rate and Characteristics of Patients Dying in Relation to COVID-19 in Italy. JAMA. 2020 Mar 23. doi: 10.1001/jama.2020.4683.
2. Zhou F, Yu T, Du R, Fan G, Liu Y, Liu Z, Xiang J, Wang Y, Song B, Gu X, Guan L, Wei Y, Li H, Wu X, Xu J, Tu S, Zhang Y, Chen H, Cao B. Clinical course and risk factors for mortality of adult inpatients with COVID-19 in Wuhan, China: a retrospective cohort study. Lancet. 2020 Mar 11. Pii: S0140-6736(20)30566-3. Doi: 10.1016/S0140-6736(20)30566-3. [Epub ahead of print]
3. Fanelli V et al. Acute respiratory distress syndrome: new definition, current and future therapeutic options. J Thorac Dis 2013;5(3):326-334. doi: 10.3978/j.issn.2072-1439.2013.04.05
4. Griffiths M et al. Guidelines on the management of acute respiratory distress syndrome. BMJ Open Resp Res 2019;6:e000420. doi:10.1136/bmjresp-2019-000420
5. Schmidt J et al. Flow-controlled ventilation during ear, nose and throat surgery: A prospective observational study. European Journal of Anaesthesiology (EJA). 2019 Feb 5.
6. Schmidt J et al. Flow-Controlled Ventilation Attenuates Lung Injury in a Porcine Model of Acute Respiratory Distress Syndrome: A Preclinical Randomized Controlled Study. Crit Care Med 2020; 48:e241–e248 DOI: 10.1097/CCM.0000000000004209
7. Bergold et al. Flow-controlled ventilation – A novel approach to treating severe acute respiratory distress syndrome. Poster WAMM, 2019

8. Barnes and Enk. Ventilation for low dissipated energy achieved using flow control during both inspiration and expiration. Trends in Anaesthesia and Critical Care 24 (2019); 5-12
9. Schmidt et al. Improved lung recruitment and oxygenation during mandatory ventilation with a new expiratory ventilation assistance device: Eur J Anaesthesiol. 2018 Oct;35(10):736-744

15. Appendices

- Poster Bergold (ref 7)
- Patient information and consent form
- Data collection sheet
- Delegate list
- Budget sheet
- SOFA-score table
